# The lncRNA DANCR promotes development of atherosclerosis by regulating the miR-214-5p/COX20 signaling pathway

**DOI:** 10.1186/s11658-022-00310-2

**Published:** 2022-02-17

**Authors:** Ruolan Zhang, Yuming Hao, Jinrong Zhang

**Affiliations:** 1 Department of Cardiology, Harrison International Peace Hospital, No. 180 Renmin Road, Hengshui City, 053000 Hebei Province People’s Republic of China; 2 Department of Cardiology, Second Affiliated Hospital of Hebei Medical University, Shijiazhuang City, 05000 Hebei Province People’s Republic of China

**Keywords:** Atherosclerosis, DANCR, miR-214-5p, COX20

## Abstract

**Background:**

Although long non-coding RNA differentiation antagonizing non-protein coding RNA (DANCR) has been reported to be involved in atherosclerosis (AS) development, its specific mechanism remains unclear.

**Methods:**

DANCR expression levels in blood samples of AS patients and oxidized low-density lipoprotein (ox-LDL) treated vascular smooth muscle cells (VSMCs) and human umbilical vein endothelial cells (HUVECs) were detected by quantitative real-time polymerase chain reaction (qRT-PCR). The small interfering RNA targeting DANCR (si-DANCR) was used to silence DANCR expression. Cell viability was assessed by CCK-8 assay. Cell apoptosis was evaluated by flow cytometry. Levels of inflammatory cytokines, anti-oxidative enzyme superoxide dismutase (SOD) activity, and malonaldehyde (MDA) were detected by specific commercial kits. An animal AS model was established to confirm the role of DANCR/microR-214-5p/COX20 (the chaperone of cytochrome *c* oxidase subunit II COX2) in AS development.

**Results:**

DANCR was significantly increased in the blood samples of AS patients and ox-LDL treated VSMCs and HUVECs. DANCR downregulation obviously increased viability and reduced apoptosis of ox-LDL-treated VSMCs and HUVECs. Meanwhile, DANCR downregulation reduced the levels of inflammatory cytokines, including interleukin (IL)-6 (IL-6), IL-1beta (IL-1β), IL-6 and tumor necrosis factor (TNF)-alpha (TNF-α) and MDA while increasing the SOD level in ox-LDL-treated VSMCs and HUVECs. DANCR regulated COX20 expression by acting as a competing endogenous RNA (ceRNA) of miR-214-5p. Rescue experiments demonstrated that miR-214-5p downregulation obviously attenuated si-DANCR-induced protective effects on ox-LDL-caused endothelial injury.

**Conclusions:**

Our results revealed that DANCR promoted AS progression by targeting the miR-214-5p/COX20 axis, suggesting that DANCR might be a potential therapeutic target for AS.

**Supplementary Information:**

The online version contains supplementary material available at 10.1186/s11658-022-00310-2.

## Introduction

Atherosclerosis (AS) is a chronic inflammatory disorder and the most common leading cause of cerebrovascular diseases (CVD) [1, 2]. Its pathogenesis is not well understood, which is one of the reasons for its high mortality [3]. It has been reported that endothelial cell dysfunction is an essential process for AS progression [4]. Oxidative low-density lipoprotein (ox-LDL) is an accepted substance for mimicking AS conditions in vitro via stimulating endothelial cell dysfunction [5]. Therefore, understanding the specific molecular mechanisms involved in ox-LDL-induced endothelial cell dysfunction may contribute to developing efficient diagnostic and therapeutic targets for AS.

Long non-coding RNAs (lncRNAs), a type of RNA transcripts more than 200 nucleotides in length, have been reported to participate in various biological and pathological processes [6]. Many lncRNAs have been shown to regulate AS progression by acting as competing endogenous RNAs (ceRNAs) of microRNAs (miRNAs) [7]. For example, OIP5-AS1 reduces ox-LDL-treated HUVEC injury via miR-30c-5p-mediated NF-kappaB signaling [8]. NEAT1 regulates endothelial functions in subclinical hypothyroidism by targeting the miR-126/TRAF7 axis [9]. PCA3 reduces lipid accumulation and AS progression by regulating the miR-140-5p/RFX7/ABCA1 signaling pathway [10]. Differentiation antagonizing non-protein coding RNA (DANCR), a widely studied lncRNA, has been reported to participate in the development of various human cancers such as non-small cell lung cancer [11] and colorectal cancer [12]. Recently, Zhang et al. found that DANCR participated in AS development by regulating ABCA1 [13], indicating that DANCR plays an important role in AS progression.

MiRNAs are another type of non-coding RNAs with regulatory functions [14]. MiR-214-5p, a newly identified miRNA, has been reported to regulate the progression of human diseases such as acute kidney injury [15], bone defects [16], prostate cancer [17], and breast cancer [18]. Interestingly, previous studies reported that DANCR could act as a ceRNA of miR-214-5p to impact the development of human diseases, such as non-small lung cancer [19] and pancreatic cancer [20]. Based on this evidence, we speculated that DANCR might play a role in AS progression through modulating miR-214-5p.

COX20 is a subunit of cytochrome *c* oxidase and is also known as the chaperone of cytochrome *c* oxidase subunit II (COX2) [21]. Although the function of COX20 in AS development remains unclear, COX2 could stimulate inflammation, thereby aggravating AS progression [22]. COX2 inhibition might aid in preventing AS progression [23]. As one of the essential chaperones of COX2 [21, 24], COX20 might also play an important role in AS. Our study aimed to investigate the potential regulatory relationship between DANCR and miR-214-5p and the role of COX20 in AS.

In this study, we confirmed that DANCR was significantly upregulated in the blood samples of AS patients and ox-LDL treated VSMCs and HUVECs. DANCR negatively regulated miR-214-5p by acting as its ceRNA. Furthermore, COX20 was identified as a direct target of miR-214-5p. Both in vitro and in vivo experiments demonstrated that DANCR/miR-214-5p/COX20 closely participated in AS progression and could be a potential therapeutic target for AS treatment.

## Materials and methods

### Clinical samples

A total of 60 volunteers, including 30 AS patients and 30 healthy controls, were recruited at Harrison International Peace Hospital between 2017 and 2020. Atherosclerosis was diagnosed with transthoracic echocardiography and carotid ultrasonography. The diagnostic criteria for AS required that the main blood vessels of the coronary artery have more than 50% stenosis. Meanwhile, patients with cancers, congestive heart failure, valvular heart diseases, hematological system diseases, autoimmune diseases, and/or infection were excluded. The blood samples from 60 volunteers were collected. All patients were diagnosed for the first time without any drug treatment, and blood samples were collected at the initial diagnosis stage. Table 1 shows the clinical characteristics of AS patients.

**Table 1:** Baseline characteristics of the subjects

Variables	Control group	AS group	p value
Sample size	30	30	–
Gender, number (%)			
Female	14 (46.7)	13 (43.3)	0.605
Male	16 (53.3)	17 (56.7)	
Age (years), mean ± SD	60.54 ± 9.21	64.60 ± 8.61	0.251
BMI (kg/m^2^), media (IQR)	22.89 (1.52)	23.15 (2.53)	0.49
Diabetes, number (%)			
Yes	13 (43.3)	15 (50)	0.629
No	17 (56.7)	15 (50)	
Hypertension, number (%)			
Yes	12 (40)	18 (40)	0.602
No	18 (60)	12 (60)	
Smoke, number (%)			
Yes	14 (46.7)	15 (50)	0.667
No	16 (53.3)	15 (50)	
Alcohol, number (%)			
Yes	16 (53.3)	15 (50)	0.7
No	14 (46.7)	15 (50)	
TC (mmol/L)	1.21 ± 0.55	1.45 ± 0.65	0.209
TG (mmol/L)	4.01 ± 0.98	4.35 ± 0.73	0.299
LDL (mmol/L)	2.02 ± 0.81	2.56 ± 0.64	0.384
HDL (mmol/L)	1.19 ± 0.47	1.17 ± 0.34	0.977

Data are presented as mean ± SD. Pearson χ^2^ test. *P < 0.05 was considered statistically significant

*BMI* Body Mass Index, *TC* total cholesterol, *TG* triglycerides, *LDL* low-density lipoprotein, *HDL* high-density lipoprotein

### Cell culture and treatment

HEK-293A, VSMCs, and HUVECs were purchased from ATCC and cultured in RPMI-1640 medium (Gibco, USA) supplemented with 10% fetal bovine serum (FBS, Invitrogen, Carlsbad, USA) in a 37 °C incubator with 5% CO_2_. To mimic the atherosclerosis condition, 1 × 10^6^ cells were seeded into six-well plates. After being cultured overnight, cells were treated with 25, 50, and 100 μg/ml oxidized low-density lipoprotein (ox-LDL) (UnionBiol, Beijing, China) for 48 h, or treated with 50 μg/ml ox-LDL for 6, 12, 24, and 48 h. To explore the function of DANCR in AS, cells were treated with 50 μg/ml ox-LDL for 48 h.

### Cell transfection

MiR-214-5p mimics, miR-214-5p inhibitor and their negative controls (miR-NC and inhibitor NC), as well as the small interfering RNA targeting DANCR (si-DANCR) and the negative control si-NC, were designed by and purchased from GenePharma (Shanghai, China). The sequence of si-DANCR is 5′-UCGGAGGUGGAUUCUGU UA-3′, and the sequence of si-NC is 5′-AGCCAACTATCCCTTCAGT-3′. 1 × 10^6^ cells were seeded into six-well plates, and cell transfection was performed with 50 nM mimics/inhibitor/si-RNAs using Lipofectamine 2000 (Invitrogen), and subjected to 50 μg/ml ox-LDL for 48 h, then cells were used for the following analysis.

### CCK-8 assay

In brief, 1 × 10^4^ transfected VSMCs and HUVECs were seeded into 96-well plates and treated with 50 μg/ml ox-LDL for 48 h. Then 10 μl of CCK-8 reagent (CCK-8, Dojindo Molecular Technologies, Japan) was added, and cells were incubated for another 4 h. Finally, the absorbance at 450 nm was measured using a microplate reader.

### Apoptosis analysis

Cell apoptosis was assessed using Annexin V-fluorescein isothiocyanate (FITC) apoptosis detection kit (Beyotime, China). Briefly, 1 × 10^6^ transfected VSMCs and HUVECs were seeded into 6-well plates and treated with 50 μg/ml ox-LDL for 48 h. Cells were then incubated with 5 μl of FITC and 5 μl of PI for 20 min. The percentage of apoptotic cells was calculated by FACSCalibur (BD Biosciences, Australia).

### Quantitative real-time polymerase chain reaction (qRT-PCR)

Total RNAs were extracted from blood samples and cells using TRIzol reagent (Invitrogen). cDNAs were synthesized via reverse transcription of approximately 1.2 μg of total RNAs using PrimeScript RT Master Mix (TaKaRa, Dalian, China). The real-time PCR reactions were performed using the Thermal Cycler Dice Real-Time PCR System (TaKaRa) at 95 ℃ for 10 s followed by 45 cycles of 95 ℃ for 5 s, 60 ℃ for 10 s, and 72 ℃ for 10 s and extension at 72 ℃ for 5 min. The RNA expression was analyzed using the 2^−ΔΔCt^ method. GAPDH and U6 were used as the endogenous references for lncRNA/mRNA and miRNA, respectively. The primers are shown in Table 2.

**Table 2:** The sequences of specific primers

Gene name	Primer sequence (5ʹ to 3ʹ)
DANCR	Forward: 5ʹ-GCCACTATGTAGCGGGTTTC-3ʹ Reverse: 5ʹ-ACCTGCGCTAAGAACTGAGG-3ʹ
miR-214-5p	Forward: 5ʹ-GCCGAGTGCCTGTCTACACT-3ʹ
	Reverse: 5ʹ-CTCAACTGGTGTCGTGGA-3ʹ
COX20	Forward: 5ʹ-TCTGTTGTGGCTGGCTTTGGAC-3ʹ Reverse: 5ʹ-CTTCTCTGGCAATTCTTTCCTGG-3ʹ
GAPDH	Forward: 5ʹ-ATCCACGGGAGAGCGACAT-3ʹ Reverse: 5ʹ-CAGCTGCTTGTAAAGTGGAC-3ʹ
U6	Forward: 5ʹ-ACAGATCTGTCGGTGTGGCAC-3ʹ Reverse: 5ʹ-GGCCCCGGATTATCCGACATTC-3ʹ

### Western blot

Total proteins were extracted using RIPA lysis buffer. Equal amounts of proteins were separated by 12% SDS-PAGE and transferred onto polyvinylidene difluoride (PVDF) membranes. After being blocked, the membranes were incubated with primary antibodies against COX20 (1:1000, ab18197, Abcam), caspase 3 (1:1000, ab32147, Abcam), cleaved caspase 3 (1:1000, ab53699, Abcam), Bax (1:500, ab32503, Abcam), Bcl-2 (1:500, ab32124, Abcam) and GAPDH (1:1000, ab181602, Abcam) overnight at 4 °C. Protein signals were observed by a chemiluminescence detection system, and relative expression levels of target proteins were analyzed using Image-Pro Plus 6.0.

### Oxidative stress assay

To evaluate oxidative stress in vitro, 1 × 10^4^ transfected VSMCs and HUVECs were seeded into 96-well plates and treated with 50 μg/ml ox-LDL for 48 h. The anti-oxidative enzyme superoxide dismutase (SOD) activity and malonaldehyde (MDA) content were detected using the appropriate specific detection kits.

### ELISA assay

About 1 × 10^4^ transfected VSMCs and HUVECs were seeded into 96-well plates and treated with 50 μg/ml ox-LDL for 48 h. The levels of inflammatory cytokines including IL-1β, IL-6, and TNF-α were measured using appropriate specific commercial detection kits. The concentrations of high-density lipoprotein cholesterol (HDL-c), low-density lipoprotein cholesterol (LDL-c), and total cholesterol (TC) in mouse serum were detected using appropriate specific commercial detection kits.

### Luciferase reporter assay

The binding sites among DANCR, miR-214-5p, and COX20 were predicted by TargetScan. To confirm their binding sites, wild type (WT) and mutant (MUT) DANCR and COX20 containing miR-214-5p binding sites were synthesized by GenePharma (Shanghai, China) and cloned into the luciferase reporter vector pmirGLO (Promega, Madison, Wisconsin). The recombinant luciferase reporter vectors were co-transfected with miR-214-5p mimics or miR-NC into VSMCs and HUVECs using Lipofectamine 2000. After transfection for 48 h, the relative luciferase activities were measured using the dual luciferase reporter system.

### Adenovirus construction

The full-length DANCR and miR-214-5p were subcloned in an adenoviral shuttle vector, pAdtrace-Tox, to construct pAdtrace-DANCR or pAdtrace-miR-214-5p, which were further verified by DNA sequencing. The pAdtrace-DANCR or pAdtrace-miR-214-5p were transfected into AdEasier cells (BJ5183 cell) to generate recombinant adenovirus AAV-DANCR or AAV-miR-214-5p. The recombinant adenoviruses were amplified in HEK-293A cells, purified using CsCl_2_ banding, and dialyzed against 10 mM Tris-buffered saline with 10% glycerol. These viruses were titrated using the Adeno-X Rapid Titer kit (BD Biosciences Clontech, Palo Alto, CA, USA) according to the manufacturer’s instructions.

### Animal model

All C57BL/6J mice, including 18 ApoE^−/−^ mice and 6 normal mice (6–8 weeks, weighing 16–21 g), were purchased from Vital River Laboratory Animal Technology Co., Ltd., (Beijing, China). The animal model with AS was established by feeding with an atherogenic diet (10% cholesterol, 10% lard, 78% basal feed, and 2% cholate) for 8 weeks as previously described [25]. Normal mice in the sham group were fed with a normal diet (ND), and ApoE^−/−^ mice in the AS model group received a high-fat diet (HFD) for 12 weeks. After that, 18 model mice were injected with 200 μl of adenovirus (1 × 10^10^ pfu/ml) overexpressing miR-214-5p and COX20 (AAV-miR-214-5p and AAV-COX20) through tail vein injection twice a week for the last 4 weeks. The empty adenoviruses without any exogenous gene were used as the negative control (AAV-mock). Finally, mice were anesthetized by intraperitoneally injecting 3% pentobarbital sodium (Sigma-Aldrich). After collecting blood samples, mice were sacrificed for the subsequent pathological morphologic analysis. Each group included six mice.

### Hematoxylin and eosin (H&E) staining assay

After sacrifice, the aortas of mice were removed and sectioned into 5 μm thick aorta sections. The morphological changes were observed after H&E staining as described previously [26]. The images were captured using an optical microscope (Olympus) at 400× magnification.

### Statistical analysis

Data were presented as mean ± SD, and statistical analysis was performed using SPSS 18.0 software. Each experiment was repeated three independent times. The difference between two groups was determined by Student’s t test or one-way ANOVA. p < 0.05 was considered as the significance threshold.

## Results

### DANCR was significantly upregulated in AS

**Fig. 1:**
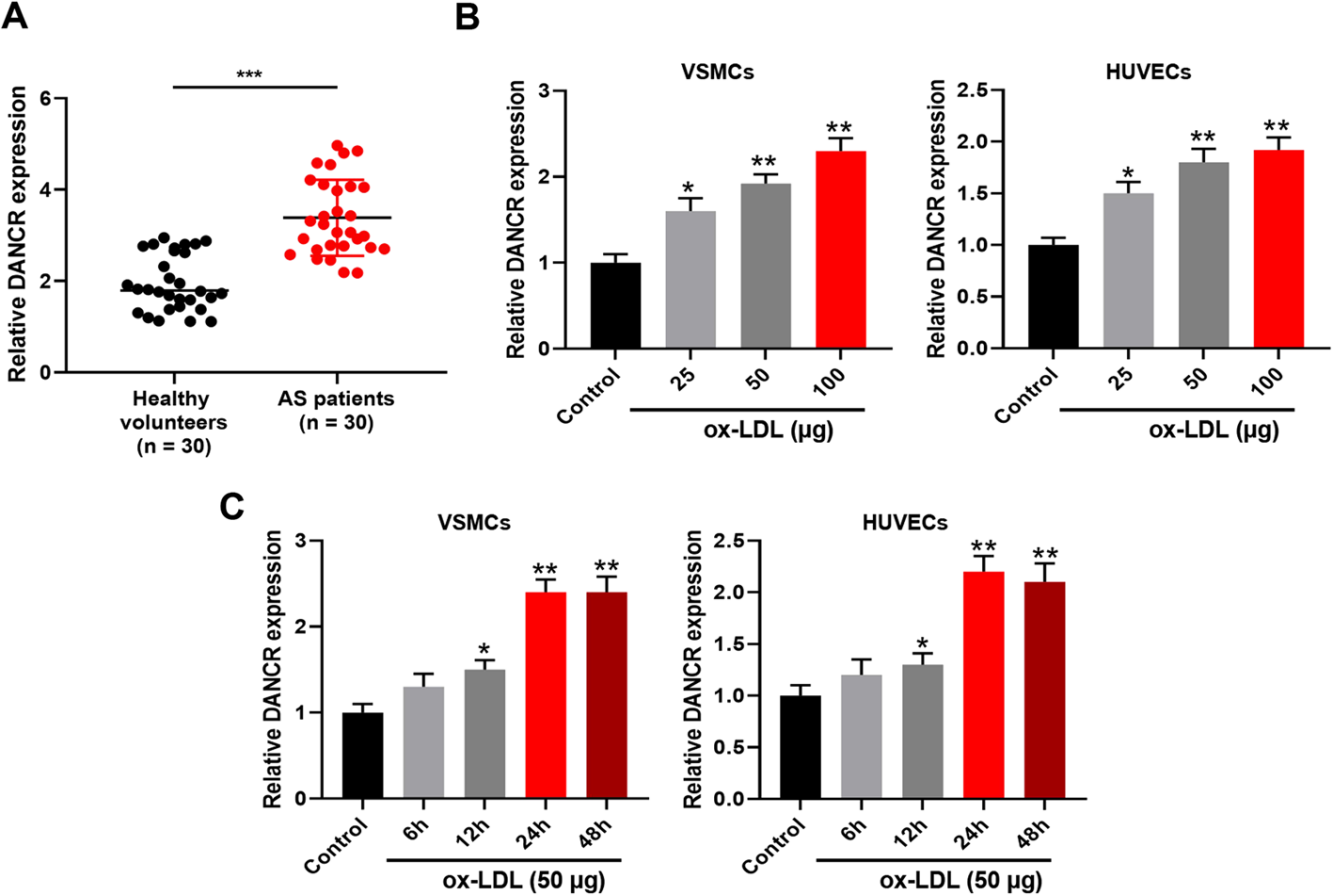
DANCR was significantly upregulated in AS. **A** DANCR expression in blood samples of AS patients was evaluated by qRT-PCR. **B** VSMCs and HUVECs were subjected to ox-LDL (25, 50, and 100 μg/ml) treatment for 48 h. DANCR expression was evaluated by qRT-PCR. **C** VSMCs and HUVECs were subjected to 50 μg/ml ox-LDL treatment for different times. DANCR expression was evaluated by qRT-PCR. Each experiment was repeated three independent times. *p < 0.05, **p < 0.01, and ***p < 0.001

The clinical characteristics were not significantly different between AS patients and healthy controls (Table 1). However, DANCR was significantly upregulated in the blood samples of AS patients (Fig. 1A). To confirm this finding, we stimulated VSMCs and HUVECs with ox-LDL (25, 50, and 100 μg/ml) for 48 h and found that DANCR was significantly upregulated in ox-LDL treated cells (Fig. 1B). Later, 50 μg/ml ox-LDL was selected for the subsequent experiments. Figure 1C showed that DANCR was obviously upregulated in 50 μg/ml treated VSMCs and HUVECs. These results suggested that DANCR might play a potential role in AS.

### DANCR downregulation ameliorated ox-LDL-induced endothelial injury in vitro

**Fig. 2:**
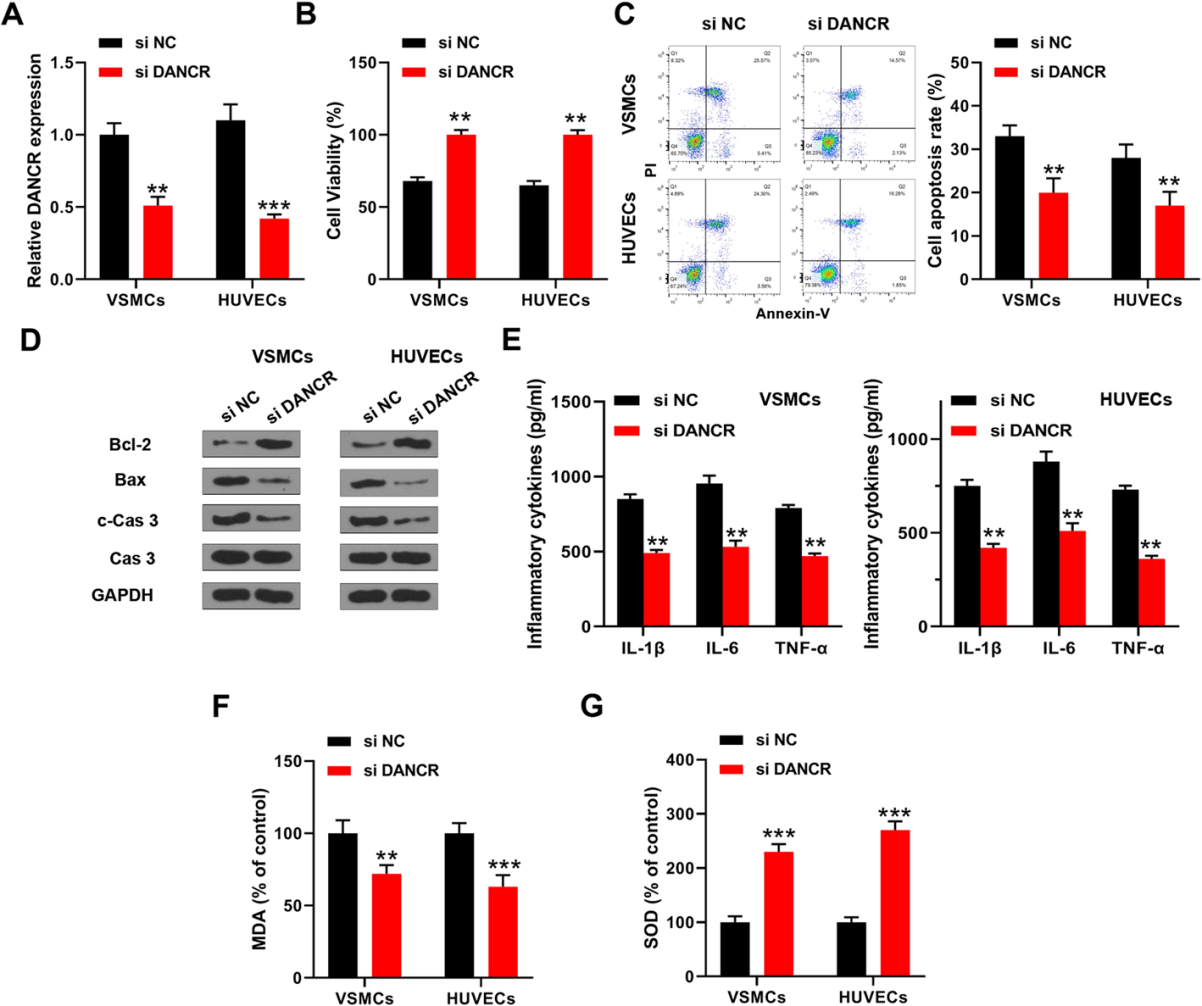
DANCR downregulation ameliorated ox-LDL-induced endothelial injury in vitro. VSMCs and HUVECs were transfected with si-DANCR or si-NC and then treated with 50 μg/ml ox-LDL for 48 h. **A** DANCR expression was detected by qRT-PCR. **B** CCK-8 assay. **C** Flow cytometry assay. **D** Expression of apoptosis-related proteins was detected by Western blot. The original images are shown in Additional file 1. **E** Expression of inflammatory cytokines was detected by ELISA assay. **F**, **G** Levels of MDA (**F**) and SOD (**G**) were evaluated by specific detection kits. Each experiment was repeated three independent times. **p < 0.01 and ***p < 0.001

VSMCs and HUVECs were transfected with small interfering RNA targeting DANCR (si-DANCR) and then treated with 50 μg/ml ox-LDL for 48 h. The transfection efficiency was confirmed by qRT-PCR (Fig. 2A). Si-DANCR significantly increased viability and reduced apoptosis of ox-LDL-treated VSMCs and HUVECs compared with si-NC (Fig. 2B, C). The levels of apoptosis-related proteins were detected by Western blot, and the results showed that si-DANCR obviously increased the Bcl-2 level, while it reduced Bax and cleaved caspase 3 levels in ox-LDL-treated VSMCs and HUVECs (Fig. 2D). In addition, si-DANCR significantly reduced the levels of inflammatory cytokines (IL-1β, IL-6, and TNF-α) and MDA, while increasing the SOD level in ox-LDL-treated VSMCs and HUVECs (Fig. 2E–G). These results indicated that DANCR downregulation ameliorated ox-LDL-induced endothelial injury in vitro.

### DANCR served as a ceRNA of miR-214-5p

**Fig. 3:**
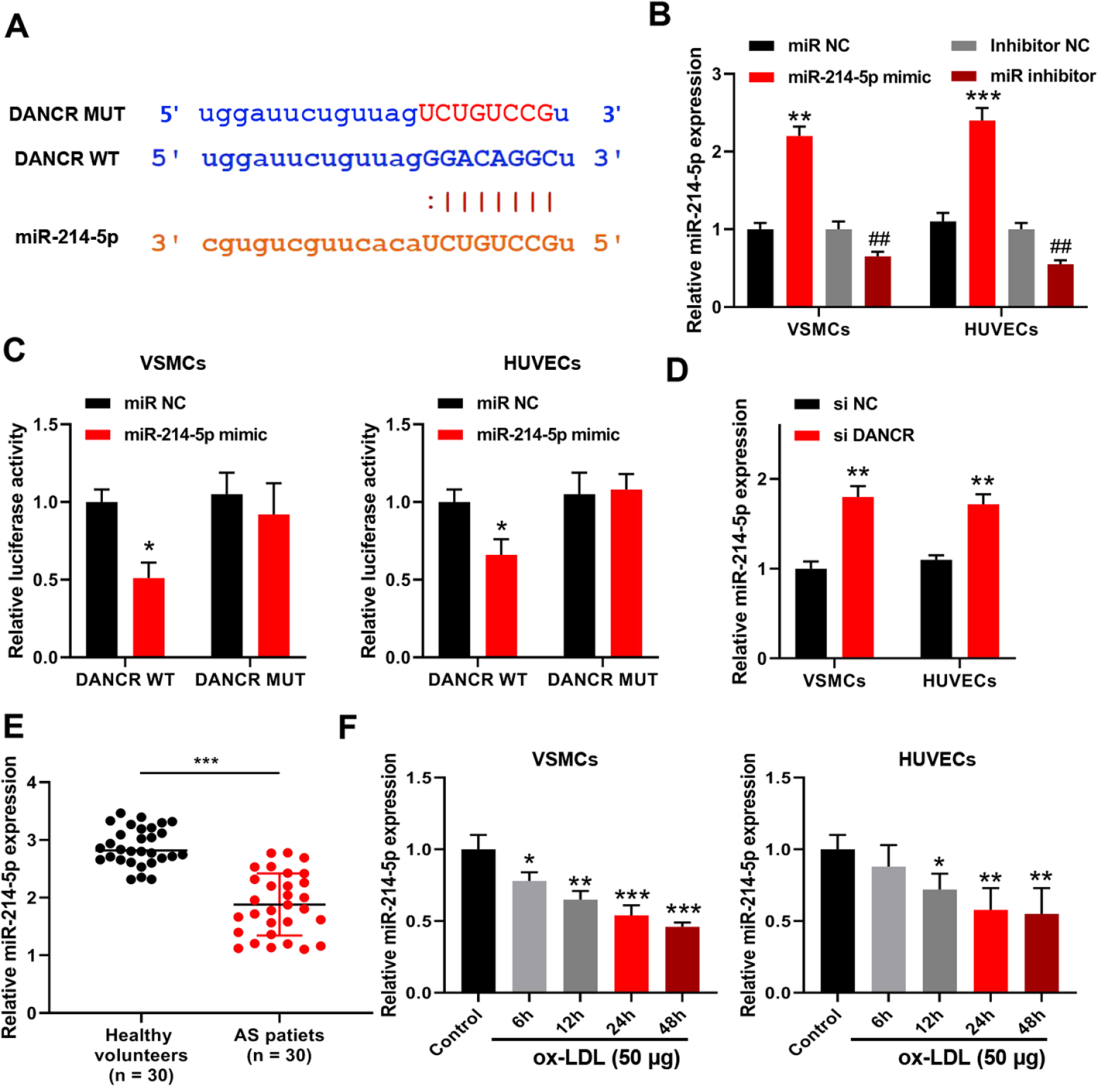
DANCR served as a ceRNA of miR-214-5p. **A** The binding site between DANCR and miR-214-5p was predicted by TargetScan. **B** MiR-214-5p mimics and inhibitor were transfected into VSMCs and HUVECs, and the transfection efficiencies were confirmed by qRT-PCR. **C** Luciferase reporter assay. **D** VSMCs and HUVECs were transfected with si-DANCR or si-NC, and miR-214-5p expression was detected by qRT-PCR. **E** MiR-214-5p expression in blood samples of AS patients was evaluated by qRT-PCR. **F** VSMCs and HUVECs were subjected to 50 μg/ml ox-LDL for different times. MiR-214-5p expression was evaluated by qRT-PCR. Each experiment was repeated three independent times. *p < 0.05, and **p < 0.01 vs. miR-NC or control group; ^##^p < 0.01 vs. inhibitor NC group

TargetScan was used to predict the binding site between DANCR and miR-214-5p, and the results are shown in Fig. 3A. To determine their binding relationship, miR-214-5p mimics and inhibitor were transfected into VSMCs and HUVECs, and the transfection efficiencies were confirmed by qRT-PCR (Fig. 3B). Luciferase reporter assay was performed, and the results indicated that miR-214-5p mimics significantly reduced the relative luciferase activity of DANCR WT compared with miR-NC in both VSMCs and HUVECs but had no effect on DANCR MUT (Fig. 3C). Meanwhile, si-DANCR significantly increased miR-214-5p expression in VSMCs and HUVECs compared with si-NC (Fig. 3D). Moreover, miR-214-5p was markedly downregulated in blood samples of AS patients compared with that in healthy volunteers (Fig. 3E). In addition, Fig. 3F showed a time-dependent reduction of miR-214-5p in 50 μg/ml ox-LDL-treated VSMCs and HUVECs. These results suggested that DANCR might serve as a ceRNA of miR-214-5p.

### COX20 was a target of miR-214-5p

**Fig. 4:**
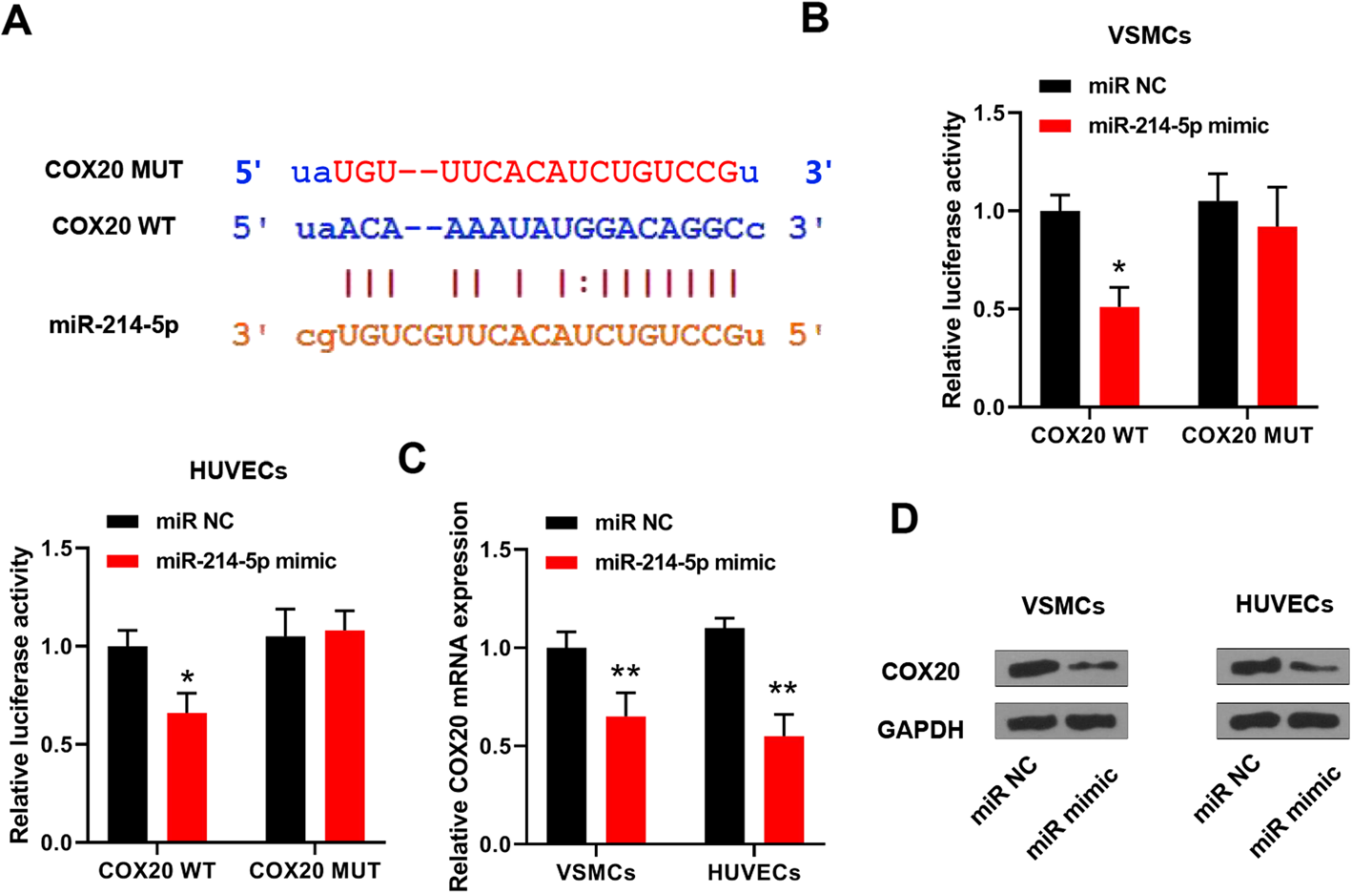
COX20 was a target of miR-214-5p. **A** The binding site between miR-214-5p and COX20 was predicted by TargetScan. **B** Luciferase reporter assay. **C**, **D** VSMCs and HUVECs were transfected with miR-214-5p mimics or miR-NC, and COX20 expression was evaluated by qRT-PCR (**C**) and Western blot (**D**). The original images are shown in Additional file 1. Each experiment was repeated three independent times. *p < 0.05, and **p < 0.01

TargetScan was applied to predict the targets of miR-214-5p, and the results suggested that COX20 might be a direct target of miR-214-5p (Fig. 4A). The results of the luciferase reporter assay showed that miR-214-5p mimics significantly reduced the relative luciferase activity of COX20 WT compared with miR-NC in both VSMCs and HUVECs but did not affect that of COX20 MUT (Fig. 4B). Meanwhile, miR-214-5p mimics significantly reduced the COX20 level at both mRNA and protein levels in VSMCs and HUVECs compared with miR-NC (Fig. 4C, D). These results suggested that COX20 might be a direct target of miR-214-5p.

### MiR-214-5p downregulation obviously attenuated the protection of si-DANCR against ox-LDL-induced endothelial injury

**Fig. 5:**
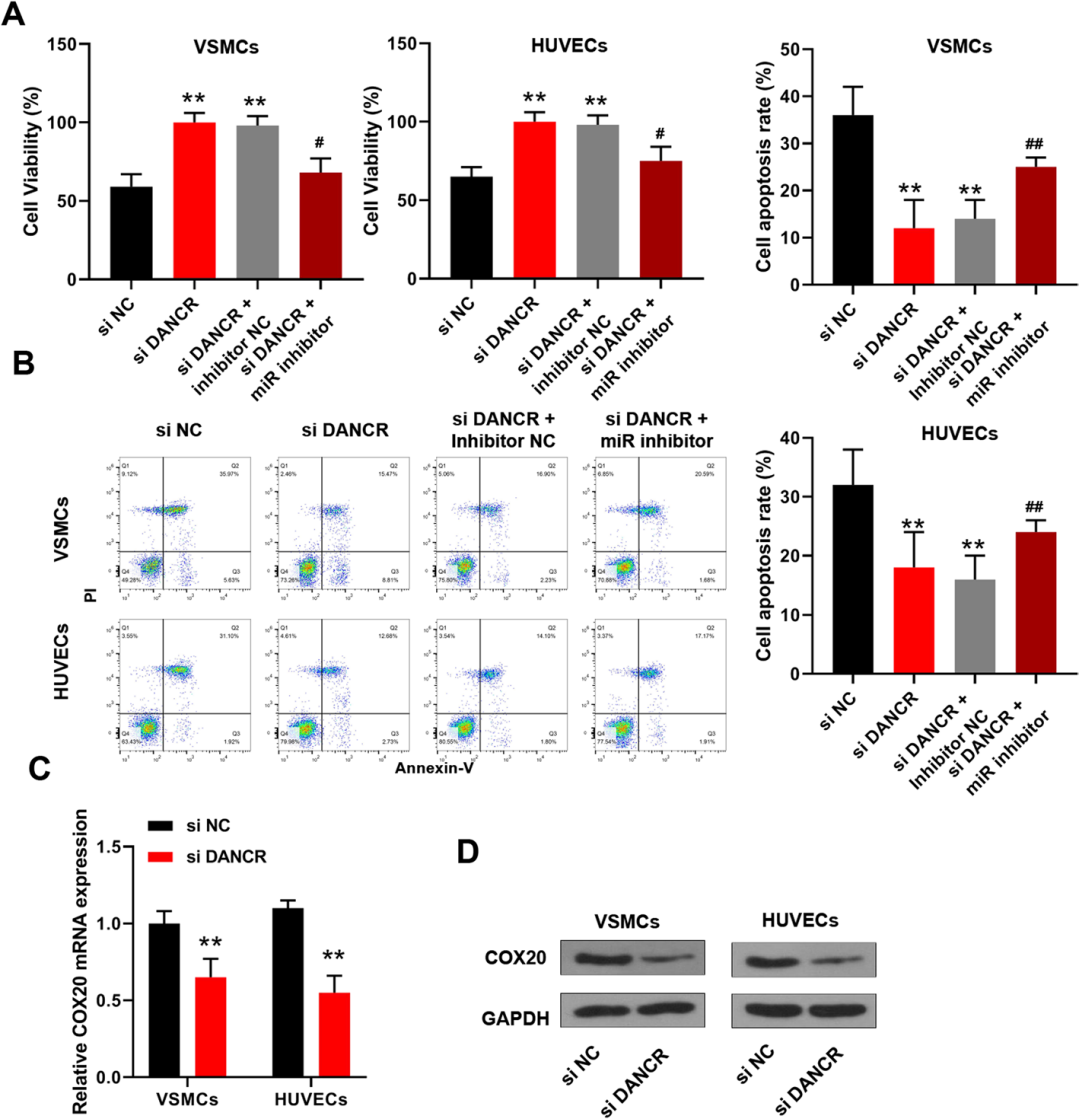
MiR-214-5p downregulation obviously attenuated si-DANCR-induced protection against ox-LDL-induced endothelial injury. **A**, **B** VSMCs and HUVECs were transfected with si-DANCR, si-NC, or co-transfected with si-DANCR and inhibitor NC, or co-transfected with si-DANCR and miR-214-5p inhibitor, and then cells were treated with 50 μg/ml ox-LDL for 48 h. **A** CCK-8 assay. **B** Flow cytometry assay. **C**, **D** VSMCs and HUVECs were transfected with si-DANCR or si-NC, and then treated with 50 μg/ml ox-LDL for 48 h. COX20 expression was evaluated by qRT-PCR (**C**) and Western blot (**D**). The original images are shown in Additional file 1. Each experiment was repeated three independent times. **p < 0.01 vs. si-NC; ^##^p < 0.01 vs. si-DANCR plus inhibitor NC group

Next, rescue experiments were performed by co-transfecting si-DANCR and miR-214-5p inhibitor into VSMCs and HUVECs. CCK-8 and flow cytometry assays of these cells treated with 50 μg/ml ox-LDL for 48 h revealed that co-transfection of si-DANCR and miR-214-5p inhibitor significantly reduced cell viability and promoted apoptosis of ox-LDL-treated cells compared with the si-DANCR plus inhibitor NC group (Fig. 5A, B). In addition, si-DANCR obviously reduced COX20 expression at both mRNA and protein levels in ox-LDL-treated VSMCs and HUVECs compared with si-NC (Fig. 5C, D). These results indicated that miR-214-5p downregulation attenuated the protective role of si-DANCR in ox-LDL-induced endothelial injury via upregulating COX20.

### DANCR/miR-214-5p/COX20 participated in the development of AS in vivo

**Fig. 6:**
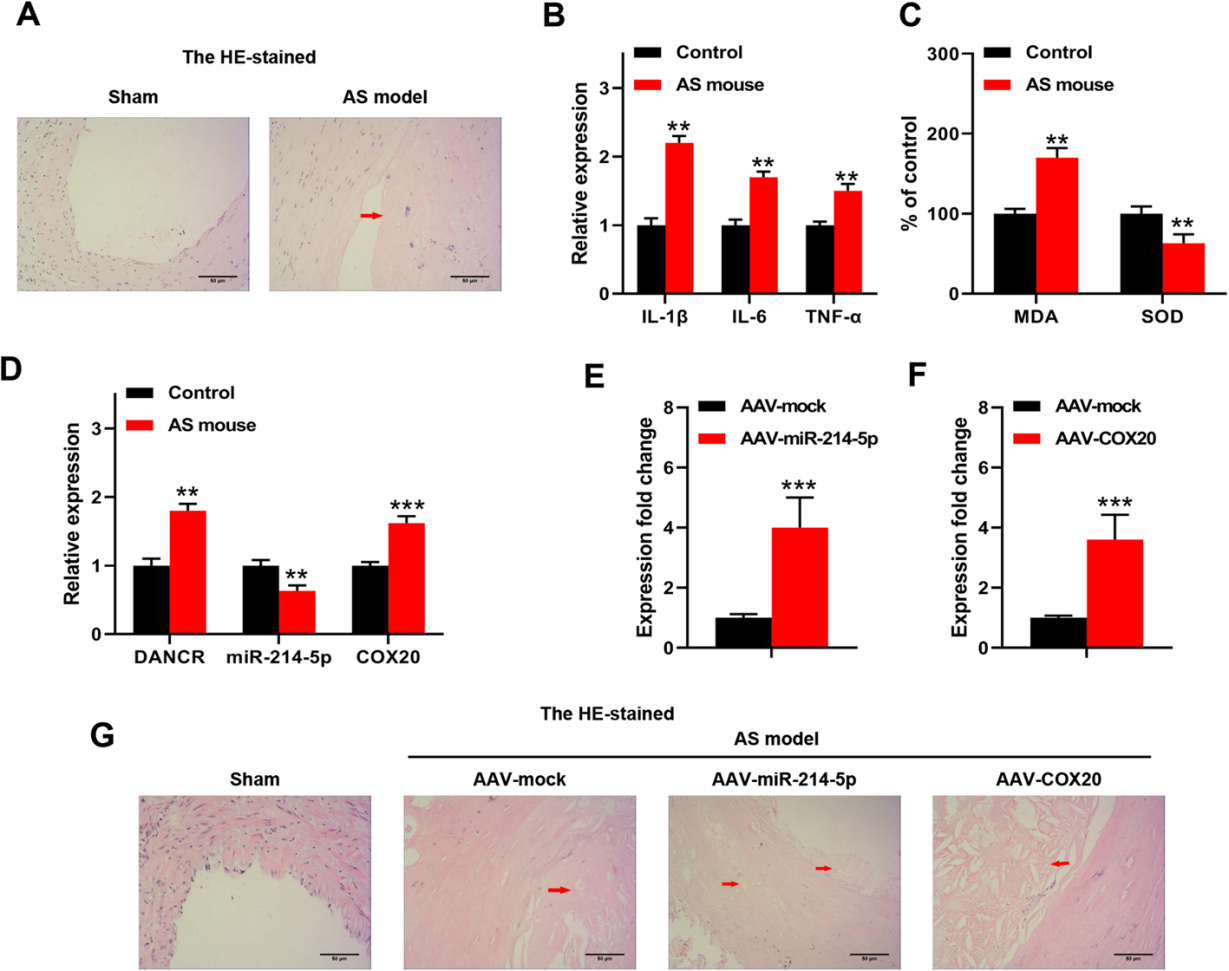
DANCR/miR-214-5p/COX20 participated in development of AS in vivo. **A** Representative micrographs of the lesion areas in the aortic sinus were observed by H&E staining at ×400 magnification, scale bar = 50 μm. **B**, **C** Levels of inflammatory cytokines (**B**), MDA, and SOD (**C**) in blood samples of AS animal model were detected by specific detection kits. **D** Expression of DANCR, miR-214-5p, and COX20 in blood samples of AS animal model was evaluated by qRT-PCR. **E**, **F** Adenovirus overexpressing miR-214-5p and COX20 were injected into mice, and expression of miR-214-5p (**E**) and COX20 (**F**) in blood samples of AS animal model was evaluated by qRT-PCR. **G** Representative micrographs of lesion areas in the aortic sinus were observed by H&E staining. ×400 magnification, scale bar = 50 μm. There were six mice in each group. Each experiment was repeated three independent times. **p < 0.01 and ***p < 0.001

To investigate the role of the DANCR/miR-214-5p/COX20 axis in vivo, an animal model with AS was established. The results of H&E staining showed that the aortic sinus plaque area was notably reduced in the AS model group compared with the control group (Fig. 6A), suggesting that the AS model was successfully established. The levels of inflammatory cytokines (IL-1β, IL-6, and TNF-α) and MDA were significantly increased, while the level of SOD was decreased in the blood samples of the AS animal model compared with the control group (Fig. 6B, C). Meanwhile, both DANCR and COX20 were significantly upregulated, while miR-214-5p was downregulated in the blood samples of the AS animal model compared with the control group (Fig. 6D). In addition, the adenovirus overexpressing miR-214-5p and COX20 were injected into mice to overexpress them, and qRT-PCR results showed that AAV-miR-214-5p significantly increased the miR-214-5p level, and AAV-COX20 significantly increased the COX20 level in the blood samples of the AS animal model compared with the control group (Fig. 6E, F) Moreover, miR-214-5p overexpression obviously reduced the lipid deposition area of the AS animal model, and COX20 overexpression further increased the lipid deposition area (Fig. 6G). In addition, lipid profiles in mice serum were examined. The results showed that the levels of HDL-c, LDL-c, and TC in the AAV-mock group were significantly higher than those in the sham group, AAV-miR-214-5p reduced the levels of HDL-c and TC compared with the AAV-mock group, while AAV-COX20 increased the levels of LDL-c and TC compared with the AAV-mock group (Additional file 1: Fig. S1). These results suggested that DANCR/miR-214-5p/COX20 closely participated in the development of AS in vivo.

## Discussion

Increasing evidence has shown an important role of lncRNA-mediated pathogenic mechanisms involved in AS progression and also highlighted the regulatory axis of the lncRNAs–miRNA–mRNA network [27, 28]. The present study revealed a regulatory axis of DANCR/miR-214-5p/COX20 in ox-LDL induced endothelial injury and in an animal model in vivo, providing a potential targeted therapeutic means for AS.

Further, we identified that DANCR acted as a ceRNA of miR-214-5p, and the luciferase reporter assay confirmed their binding relationships. Rescue experiments showed that miR-214-5p downregulation obviously eliminated the protection of si-DANCR in ox-LDL-induced endothelial injury, and also that miR-214-5p was a direct downstream miRNA of DANCR [19, 20] and mediated the function of DANCR in AS progression. It has been reported that lncRNA-mediated cell proliferation and apoptosis of smooth muscle and endothelial cells, inflammation, and oxidative stress were closely associated with the pathogenic processes during AS progression [29–31]. Hence, we explored the effects of DANCR on these important factors and found that DANCR downregulation reduced the levels of inflammatory cytokines (IL-1β, IL-6, and TNF-α) and MDA while it increased the SOD level in ox-LDL-treated VSMCs and HUVECs. These data revealed that DANCR downregulation could efficiently reduce the inflammatory response and oxidative stress during AS progression, contributing to our understanding of the pathogenic mechanism during AS development. In addition, DANCR has been identified as the ceRNA of several target miRNAs other than miR-214-5p, such as miR-185-5p [32], miR-874-3p [33], miR-1301-3p [34], miR-185-5p [35], miR-345-5p [36], miR-125b-5p [37], and miR-144-3p [38]. Since one lncRNA might target one or more downstream miRNAs [39], we hypothesized that DANCR might also have other target miRNAs that participated in the function of this lncRNA in AS progression. We will investigate this hypothesis in subsequent experiments in the future.

To further explore the specific mechanism of miR-214-5p in AS, TargetScan was applied and the prediction showed that there was a putative binding site between miR-214-5p and the 3ʹ UTR of COX20. Luciferase reporter assay determined that miR-214-5p could directly bind to the 3ʹ UTR of COX20. DANCR could positively regulate the expression of COX20. COX20 is a mitochondrial inner membrane protein and has been identified as a chaperone of COX2, a subunit of cytochrome *c* oxidase [40]. Previous studies indicated that COX20 was important for the tolerance to oxidative stress [41]. However, the role of COX20 in AS progression has not been studied. Our results showed that COX20 was significantly upregulated in the blood samples of an AS animal model, and COX20 overexpression obviously promoted the progression of AS in vivo, indicating that the role of COX20 in AS might be due to its role in oxidative stress. This study investigated the roles of DANCR, miR-214-5p, and COX20 in the proliferation, apoptosis, inflammation, and oxidative stress of ox-LDL-treated VSMCs and HUVECs, reinforcing that this axis might be a novel therapeutic target for AS.

In this study, we also observed the significant changes in the lipid profile of the experimental mice after the injections of adenovirus genetic constructs (Additional file 1: Fig. S1), and these changes might be real reasons for acceleration or deceleration of atherosclerotic lesions. In addition, considering that the main target of adenoviral gene delivery is the liver [42], the possible role of DANCR/miR-214-5p/COX20 signaling in the regulation of lipid metabolism in hepatocytes was also suggested. This conjecture should be investigated in the future.

In summary, our study demonstrated that DANCR downregulation attenuated AS progression via regulating the miR-214-5p/COX20 axis, contributing to our understanding of the pathogenesis in AS.

## Supplementary Information

**Additional file 1: Fig. S1.** Lipid profiles in mice. Serum lipid profile of C57BL/6J mice was measured. Six mice in each group. Each experiment was repeated three independent times. *p < 0.05, **p < 0.01, ***p < 0.001 vs. sham group; ^#^*p* < 0.05 vs. AAV-mock group.

## Data Availability

The data that support the findings of this study are not publicly available due to their containing information that could compromise the privacy of research participants, but are available on request from the corresponding author.

## Abbreviations

DANCR: Differentiation antagonizing nonprotein coding RNA
COX20: A subunit of cytochrome *c* oxidase and the chaperone of cytochrome *c* oxidase subunit II (COX2)
CCK-8: Cell Counting Kit-8
VSMCs: Vascular smooth muscle cells
HUVECs: Human umbilical vein endothelial cells
Ox-LDL: Oxidized low density lipoprotein
SOD: Superoxide dismutase
MDA: Malonaldehyde
H&E: Hematoxylin and eosin
