# Supplementary material for: The lncRNA DANCR promotes development of atherosclerosis by regulating the miR-214-5p/COX20 signaling pathway
Source: Cell Mol Biol Lett. 2022 Feb 17;27:15. doi: 10.1186/s11658-022-00310-2 (PMC8903577; doi:10.1186/s11658-022-00310-2)
Supplement: Supplementary file 1 — Additional file 1: Fig. S1. Lipid profiles in mice. Serum lipid profile of C57BL/6J mice was measured. Six mice in each group. Each experiment was repeated three independent times. *p < 0.05, **p < 0.01, ***p < 0.001 vs. sham group; #p < 0.05 vs. AAV-mock group. [file 11658_2022_310_MOESM1_ESM.docx]

**Supplementary Fig. S1**

**The lipid profiles in mice**

The serum was separated by centrifugation (4000 × g, 30 min) immediately after blood harvest via cardiac puncture. High-density lipoprotein cholesterol (HDL-c), low-density lipoprotein cholesterol (LDL-c), and total cholesterol (TC) were assayed using a colorimetric enzyme-linked immunosorbent assay (ELISA) method.

**Supplementary Fig. S1 The lipid profiles in mice.** Serum lipid profile of C57BL/6J mice were measured. Six mice in each group. Each experiment was repeated three independent times. * *p* < 0.05，** *p* < 0.01, *** *p* < 0.001 *vs.* the sham group; # *p* < 0.05 *vs.* the AAV-mock group.

**The original western blot gels**

**Fig. 2D for HUVECs**

**
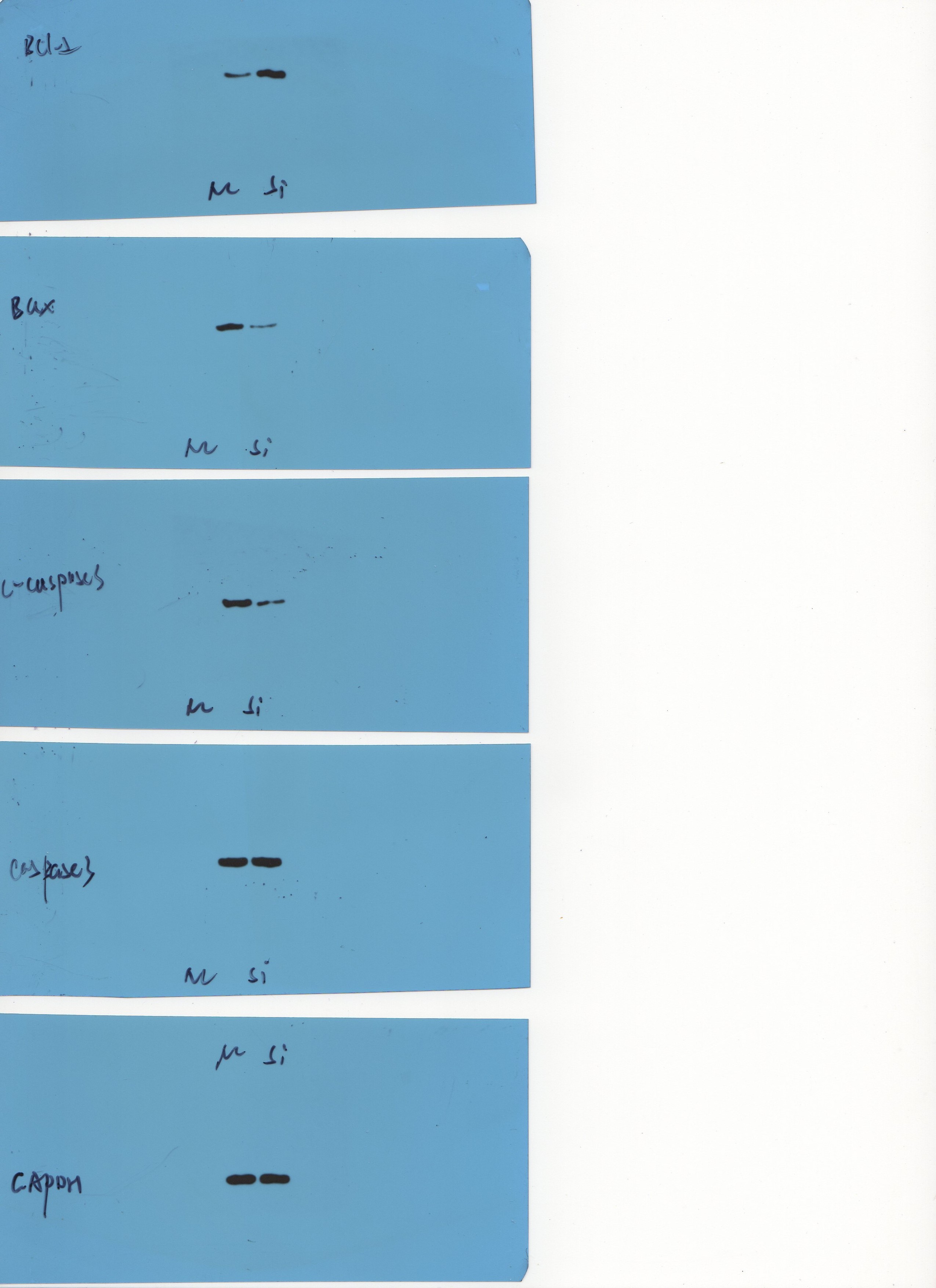
**

**Fig. 2D for VSMCs**

**
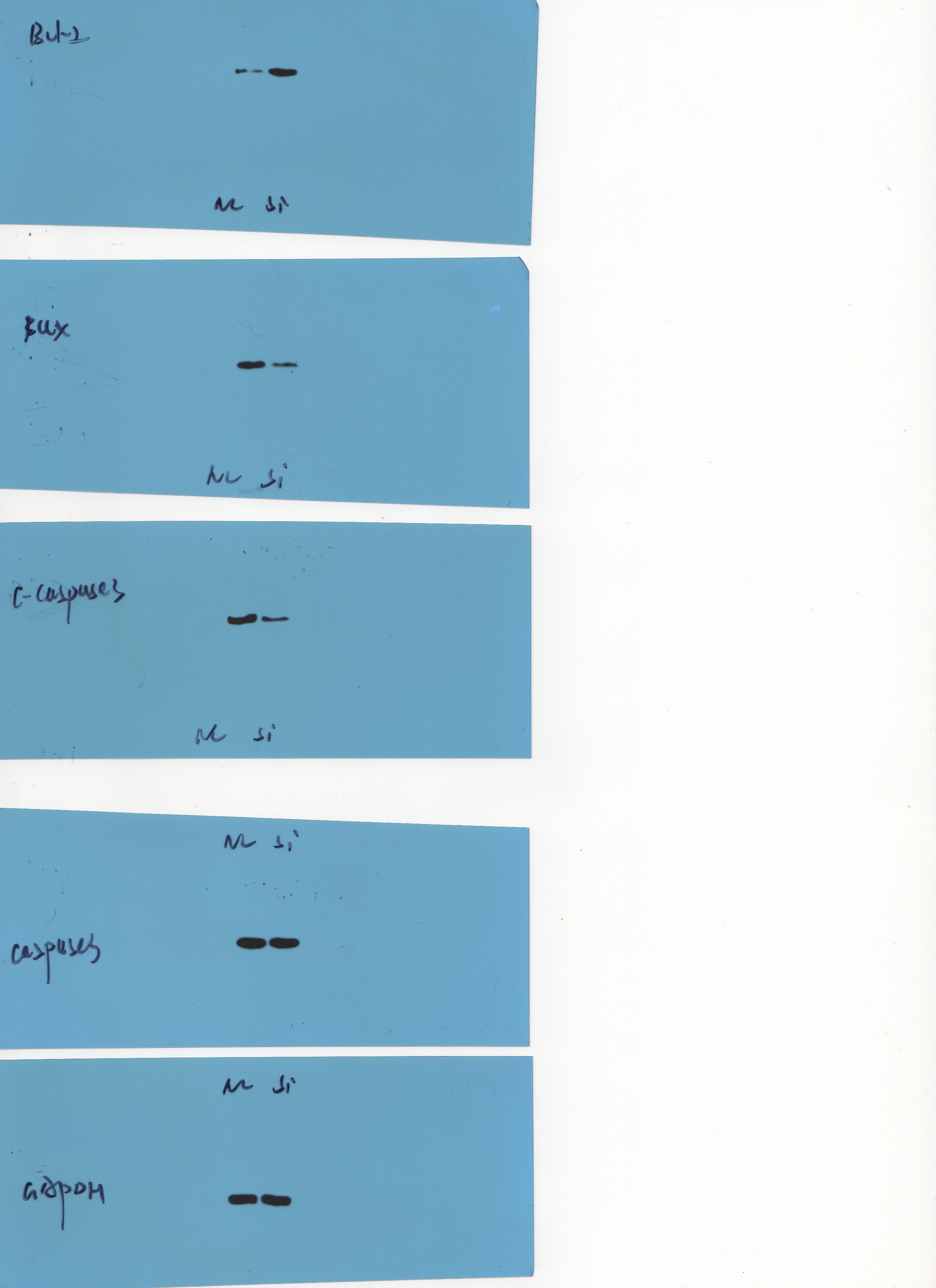
**

**Fig. 4D for HUVECs**

**
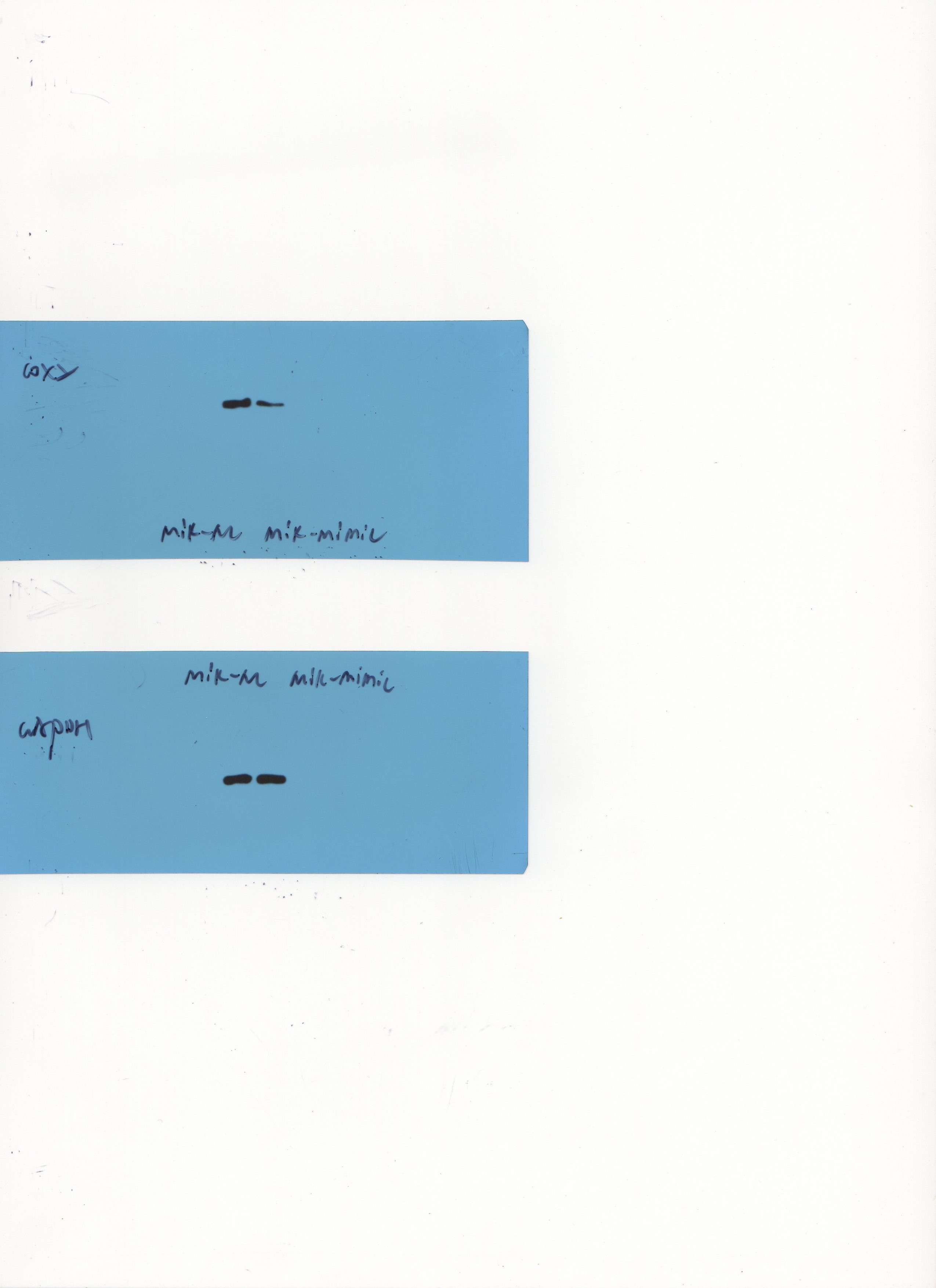
**

**Fig. 4D for VSMCs**

**
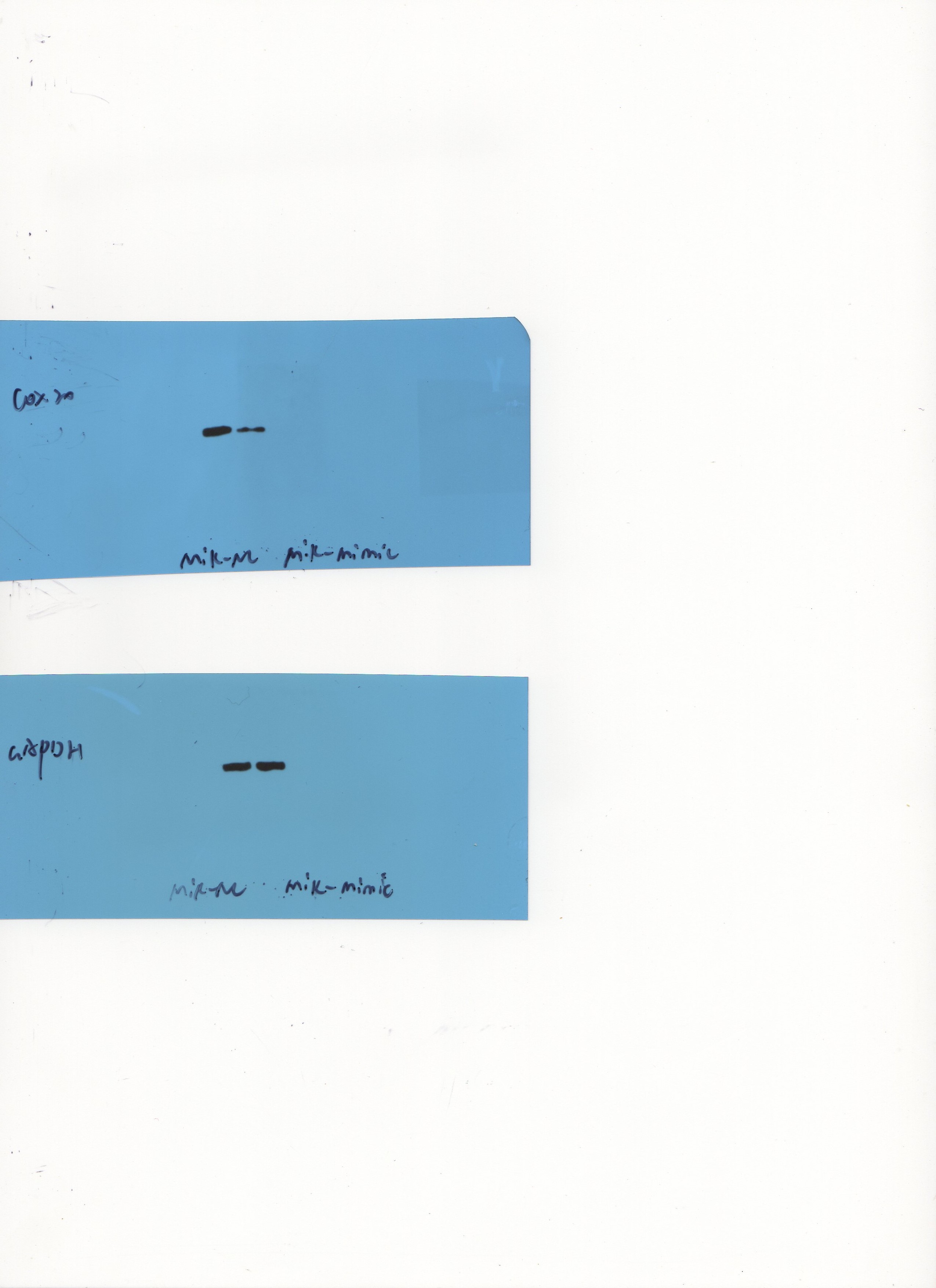
**

**Fig. 5D for HUVECs**

**
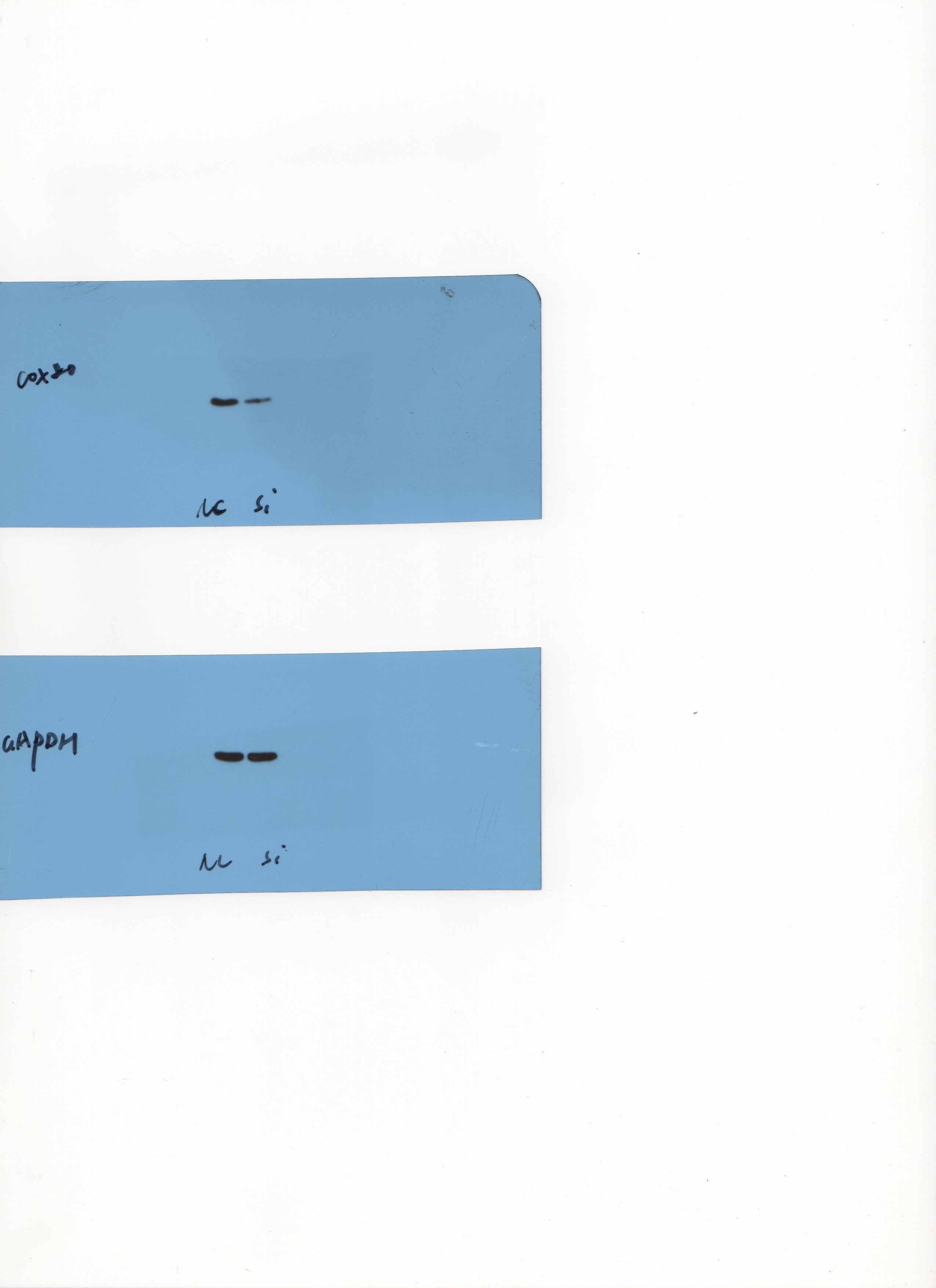
**

**Fig. 5D for VSMCs**

**
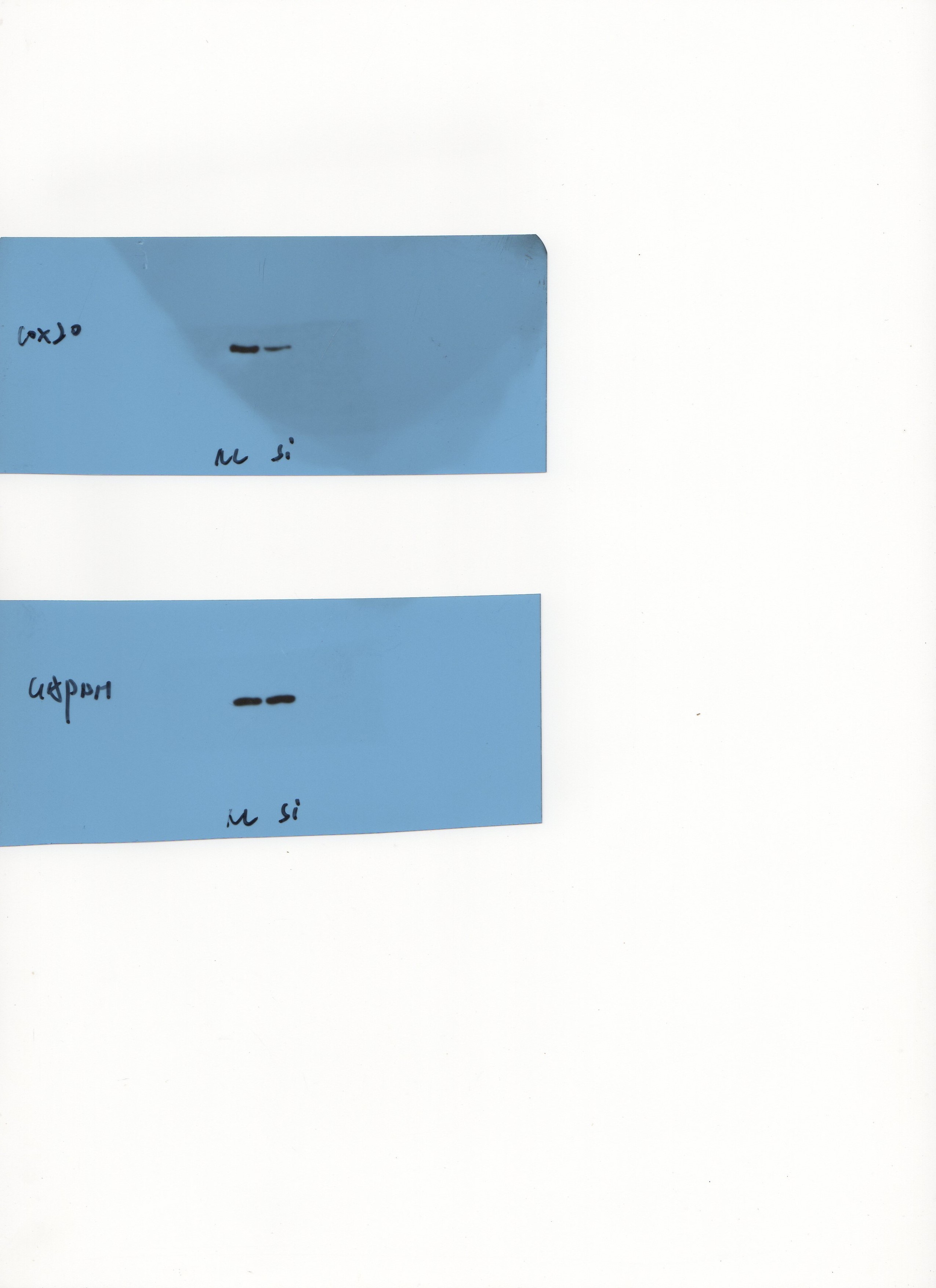
**
